# INFORMAL STUDENT CAREGIVERS: BALANCING CAREGIVING RESPONSIBILITIES AND ACHIEVING ACADEMIC SUCCESS

**DOI:** 10.1093/geroni/igac059.128

**Published:** 2022-12-20

**Authors:** Gretchen Tucker, Dana Bradley, Roberto Millar, Claudia Thorne, Christin Diehl

**Affiliations:** University of Maryland, Baltimore, Columbia, Maryland, United States; University of Maryland, Baltimore County, Baltimore, Maryland, United States; The Hilltop Institute, Baltimore, Maryland, United States; Coppin State University, Baltimore, Maryland, United States; The Hilltop Institute, Baltimore, Maryland, United States

## Abstract

According to the American Association for Retired Persons (AARP), there are five million student caregivers in the United States. Seven out of ten student caregivers say their caregiving roles affect their academic achievements. An informal (unpaid) student caregiver may provide care for a spouse, family member (excluding child care), friend, or neighbor. Student caregivers often encounter obstacles in balancing their caregiving responsibilities while trying to achieve academic success. The goal of this mixed method study is to understand the experiences of informal (unpaid) student caregivers. The aim of this study was to expand on a pilot study to increase our understanding of what challenges students face while being a caregiver and what resources are most helpful to reach their academic goals. The study was a mixed methods study consisting of a survey, which was sent to both undergraduate and graduate students, followed by one-on-one interviews with students who wanted to provide additional information about their experiences. Data was collected from two universities in the Baltimore metropolitan area, one of which is a designated historically black university and the other a minority serving university. We present findings from this research with a focus on identifying challenges and opportunities for universities to serve informal student caregivers from diverse backgrounds. More broadly, this research will contribute to the universities’ understanding of student caregivers and help identify new resources to assist students in achieving their academic goals while being a caregiver.

